# Investigation of the Association between Genetic Polymorphism of Microsomal Epoxide Hydrolase and Primary Brain Tumor Incidence

**DOI:** 10.1155/2013/189237

**Published:** 2013-12-16

**Authors:** Ali Aydin, Hatice Pinarbasi, Mustafa Gurelik

**Affiliations:** ^1^Department of Molecular Biology, Science and Art Faculty, Gaziosmanpasa University, 60150 Tokat, Turkey; ^2^Department of Biochemistry, Faculty of Medicine, Cumhuriyet University, 58100 Sivas, Turkey; ^3^Department of Neurosurgery, Faculty of Medicine, Cumhuriyet University, 58100 Sivas, Turkey

## Abstract

mEH is a critical biotransformation enzyme that catalyzes the conversion of xenobiotic epoxide substrates into more polar diol metabolites: it is also capable of inactivating a large number of structurally different molecules. Two polymorphisms affecting enzyme activity have been described in the exon 3 and 4 of the mEH gene. The hypothesis of this study is that inherent genetic susceptibility to a primary brain tumor is associated with mEH gene polymorphisms. The polymorphisms of the mEH gene were determined with PCR-RFLP techniques and 255 Turkish individuals. Our results indicate that the frequency of the mEH exon 4 polymorphism (in controls) is significantly higher than that of primary brain tumor patients (OR = 1.8, 95% CI = 1.0–3.4). This report, however, failed to demonstrate a significant association between mEH exon 3 polymorphism and primary brain tumor susceptibility in this population. Analysis of patients by both histological types of primary brain tumor and gene variants showed no association, although analysis of family history of cancer between cases and controls showed a statistically significant association (*χ* ^2^ = 7.0, *P* = 0.01). Our results marginally support the hypothesis that genetic susceptibility to brain tumors may be associated with mEPHX gene polymorphisms.

## 1. Introduction

In recent years, genetic polymorphism in a variety of xenobiotic-metabolizing enzymes (phase I and II enzymes) has been studied extensively. In general, these polymorphisms do not always lead to changes in protein expression and catalytic activities. However, the gene mutations cause alterations in expression and function of the enzymes. For this reason, several polymorphic genes, encoded for enzymes involved in phase I and II reactions, partially explain individual susceptibility to cancer. One of the enzymes is mEH, which catalyzes xenobiotic biotransformation in mammalian tissues and supports detoxification capability of the organism [1].

The mEH (EPHX1 EC 3.3.2.3) is a smooth endoplasmic reticulum enzyme that catalyzes the hydrolysis of epoxides into *trans*-dihydrodiols and is responsible for the detoxification of PAH [2, 3]. Two variant EPHX1 alleles have been associated with altered mEH activity, which is a substitution of histidine for tyrosine at residue 113 (exon 3 polymorphism); this decreases mEH activity by approximately 40%, whereas substitution of arginine for histidine at residue 139 (exon 4 polymorphism) increases enzyme activity by approximately 25% [4]. These polymorphisms also tend to affect the stability of the mEH protein.

An epoxide is a three-membered cyclic ether, while its ring system is highly strained, and its oxygen-carbon bond is polarized. Some reactive epoxides are responsible for electrophilic reactions, with critical biological targets such as DNA and protein, leading to mutagenic, toxic, and carcinogenic effects [5]. The mEH is an important enzyme in the metabolism of environmental and man-made contaminants, as it can metabolize a broad array of epoxide-containing compounds. These include aliphatic epoxides (butadiene oxide) and polyaromatic oxides (phenanthrene oxide) [6–8].

The development of disease is an outcome of interactions between human genes and the environment. Therefore, genetic variability may be responsible for individual susceptibility to carcinogenic factors in primary brain tumors. Brain tumor etiology indicates that certain occupations involving exposure to polycyclic aromatic hydrocarbons (or other aromatic hydrocarbons) may be associated with increased risk [9]. Viral agents, household chemicals, and certain foods have not been established as causal. In addition, little is known about the interaction of genetic factors and environmental toxins in the genesis of brain tumors [10].

A number of investigations have been conducted to examine the potential association of the mEH genotype with altered susceptibility to disease incidence. These investigations yield highly confusing results. However, negative or positive results have been observed in similar studies for genotypes that involve different genes. From this perspective, Badr El-Din et al. found benign brain tumors that showed a significantly higher frequency of IL-10-1082 A/A and IL-6-174 C/C homozygous genotypes, compared to controls [11]. The *SOD3* IVS1+186C>T polymorphism is associated with a significantly increased risk of meningioma, while a possible increased risk of glioma was observed by Rajaraman et al. [12]. On the other hand, Sima et al. found no significant association between the GSTM1 and GSTT1 homozygous deletion polymorphisms and risk of brain tumors [13]. In some studies, EPHX1 high-activity alleles have been associated with increased risk of various types of cancer [14]. Some studies also found that the variant EPHX1 Arg139 allele and GSTP1*C allele confer protection against solvent-induced encephalopathy [15].

The hypothesis of this study is that inherent genetic susceptibility to primary brain tumor is associated with mEH gene polymorphisms. To investigate this, we analyzed epidemiologic and molecular data from a case-controlled study in the Turkish population. We believe that there is a great need for large and well-designed epidemiological studies of potential genetic and environmental risk factors.

## 2. Materials and Methods

### 2.1. Study Population

In this study, 255 Turkish people were assessed. Cases included brain tumor patients (*n* = 105) who attended the Neurosurgery Department at Cumhuriyet University Hospital in Sivas. Only those patients with newly diagnosed primary brain tumors (without any previous radiotherapy and chemotherapy) were included in the study. There was no sex, age, or tumor type restriction. The diagnosis of brain tumor was histologically confirmed, and tumor types were classified according to WHO guidelines. Age- and sex-matched controls were recruited mainly from patients (without any previous cancer diagnosis, including radiotherapy or chemotherapy) at the same hospital (*n* = 150). All study subjects agreed to participate and filled out a short questionnaire about occupation, tobacco use, alcohol consumption, and a family history of cancer. The local university ethics committee on human research approved the study.

### 2.2. Laboratory Analysis

Five mL peripheral blood samples were collected to citrate containing tubes from all subjects. DNA was extracted from whole blood by a salting out procedure when the samples reached the laboratory.

### 2.3. Determination of mEH3 and mEH4 Genotyping

Two sequence variants of the EPHX1 gene (exon 3 and 4) were detected with the PCR and RFLP assay [16]. The target DNA sequence in EPHX1 gene exon 3 was amplified with primers *F *5′* GGG GTC CTG AAT TTT GCT CC *3′ and *R *5′* CAA TCT TAG TCT TGA AGT GAC GGT *3′, and in EPHX1 gene exon 4 the primers used were *F *5′* GGG GTA CCA GAC CTG ACC GT *3′ and *R *5′* AAC ACC GGG CCA CCC TTG GC *3′.

PCR was carried out in a volume of 50 *μ*L with 50 ng of DNA, 50 pmol of exon 3 or 4 primer, 5 *μ*L dNTPs, 4 *μ*L MgCI_2_, 5 *μ*L PCR buffer, and 0.5 *μ*L *Taq* DNA polymerase. The amplification of exon 3 was an initial denaturation at 95°C for 7 min followed by 35 cycles of denaturation at 95°C for 1 min, annealing at 53°C for 1 min, extension at 72°C for 1 min, and final extension at 72°C for 10 min. The amplification of exon 4 was an initial denaturation at 94°C for 5 min, followed by 35 cycles of denaturation at 94°C for 30 s, annealing at 63°C for 30 s, extension at 72°C for 45 s, and final extension at 72°C for 5 min. Amplified products (exon 3: 163 bp, exon 4: 357 bp) were resolved by using 1.5% wt/vol agarose gels with ethidium bromide.

### 2.4. RFLP Analysis of EPHX1 Gene Try/His 113 Variant

Following overnight digestion of the 14 *μ*L PCR product (163 bp) with 10 U *PsyI*, these products were resolved with 2.5% agarose gel containing ethidium bromide. Following digestion, the homozygous wild-type (Try 113) was identified by two DNA bands (140 and 23 bp), whereas the homozygous mutant allele (His 113) produced one band (163 bp), and the heterozygotes displayed a combination of all three (163, 140, and 23 bp) DNA fragments.

### 2.5. RFLP Analysis of EPHX1 Gene His/Arg 139 Variant

Again, after overnight digestion of the 14 *μ*L PCR product (357 bp) with 10 U *RsaI*, the digested products resolved with 2.5% agarose gel containing ethidium bromide. After digestion, homozygous wild-type (His 139) was identified by two DNA bands (295 and 62 bp), whereas the homozygous mutant allele (Arg 139) produced three bands (174, 121, and 62 bp); the heterozygotes displayed a combination of all four bands (295, 174, 121, and 62 bp) DNA fragments.

### 2.6. Statistical Analysis

The distribution of the mEPHX genotypes, tobacco use, and family history of cancer between cases and controls was evaluated using the *χ* ^2^ test or Fisher's exact test (when the expected number in any cell was less than five). In addition, the association between histopathologic type of brain tumor and mEPHX genotypes was evaluated using the *χ* ^2^ test. The strength of the association was assessed for each polymorphism individually with OR and 95% CI from logistic regression models. All analyses were conducted using the Statistical Package for Social Sciences Program (SPSS, version 14).

## 3. Results

In this study, samples were collected from 150 controls (75 for males and 75 for females) and 105 cases (55 for males and 50 for females) of primary brain tumor patients.  Table 1 shows the characteristics of the study population. The mean age of male and female patients was 49.6 ± 16.3 and 47.5 ± 17.6, respectively. The mean age of male and female controls was 50.0 ± 7.2 and 45.4 ± 15.8, respectively. No significant difference in the proportion of smokers was observed between case (25%) and control (36%) groups. The family history of cancer was more prevalent in cases (6%) than in controls (16%).

There were also no statistically significant differences between cases and controls in smoking status (*χ* ^2^ = 3.5, *P* = 0.1) (Table 2).

However, there was a statistically significant association found between case and control groups for family history of cancer (*χ* ^2^ = 7.0, *P* = 0.01) (Table 3).

The mEPHX exon 3 genotype distributions in controls and cases can be seen in Table 4. There was no significant association between mEPHX exon 3 genotype and primer brain tumor (*χ* ^2^ = 0.05, *P* = 0.8, Crude OR = 1.0, 95% CI = 0.6−1.7) (Table 4). We evaluated only 102 cases for exon 3 because there were no PCR products of the three cases of exon 3.

The mEPHX exon 4 genotype distributions in controls and cases are given in Table 4. There was a significant association between mEPHX exon 4 genotype and primer brain tumor incidence (*χ* ^2^ = 4.3, *P* = 0.03, Crude OR = 1.8, 95% CI = 1.0−3.4) (Table 4).

The polymorphisms in the mEH exon 4 and 3 were in HWE (Table 5).

We subdivided patients according to histopathologic type of brain tumor, so that we could evaluate the association between the polymorphisms of mEPHX and specific types of primer brain tumors. Histopathologic data were available for 102 cases: 29% were meningioma, 38% were astrocytoma, 9% were hypophysis adenoma, and 24% were other types (craniopharyngioma, acoustic neuroma, medulloblastoma, and colloid cyst) of exon 3. Histopathologic data were available for 105 cases: 30% were meningioma, 37% were astrocytoma, 9% were hypophysis adenoma, and 24% were other types in exon 4. There was no statistically significant association between mEPHX gene variants and tumor types (Table 6).

## 4. Discussion

Cancer is the most common cause of death after diseases of the cardiovascular system. Brain tumor is a predominant problem worldwide, in terms of both incidence and prevalence, and is a leading cause of tumor death. Brain tumors affected more than 18,300 American males in 2003, and the mortality rate was 72% (i.e., 13,100 men died of brain tumor in the same year). The most common brain tumors are gliomas, which originate in the supportive glia tissue: astrocytomas arise from astrocytes [17]. Brain tumors can strike anyone, but risk increases with age, certain lifestyles, and quality of the environment. Genetic factors that contribute to brain tumor etiology are poorly understood. Exposure to chemical, physical, and biologic agents is considered to at least be a factor in the development of a brain tumor. Our study was based on the hypothesis that the risk of a primary brain tumor would be modified by genetic polymorphism in enzymes involved in the metabolism of common chemicals, such as PAH. Gene products show genetic variability in activity among individuals. Genetic variability may be responsible for individual susceptibility to carcinogenic factors in brain tumors, as well as other tumors such as lung, ovarian, and colorectal [18–20]. The most consistent epidemiologic finding for brain tumors is that primary ones are more common among males, whereas meningeal tumors are more common among females [21]. Cultural, ethnic, and geographic differences in risk factors may also influence differences in tumor incidence; the incidence of malignant brain tumors in Japan is less than half that in Northern Europe. In the United States, gliomas affect a greater proportion of Caucasians than African-Americans, but the incidence of meningioma is nearly equal [22]. There have not been any studies to date in the literature that indicate an association between brain tumors and mEPHX gene polymorphism.

In this study, 60% of meningeal tumors were more common among female patients, such that these results were consistent with the literature. We found no association between primary brain tumor incidence and mEPHX exon 3 polymorphism (*χ* ^2^ = 0.05, *P* = 0.8, OR = 1.0, 95% CI = 0.6−1.7) (Table 4). Some studies found a slight association between primary brain tumor and mEPHX exon 3 gene polymorphism. For example, De Roos et al. reported a poorly increased risk associated with EPHX1 113: His/His for glioma was predominant with elevated risks among older subjects (>50), females, and lifelong-smokers. These patterns were not clear for meningioma or acoustic neuroma [23]. According to several studies, an increased risk of cancer is associated with lower activity of the His/His protein product; nevertheless, EPHX1 high-activity alleles have been associated with an increased risk for various types of cancer [24–27]. Gene variants may play a more complex role in human carcinogenesis as well. However, there may be other mechanisms that affect brain carcinogenesis, such as tumor suppressor gene with defects (PTEN, RB1, CDKN2A, CDKN2B, P^14^ARF, P53) and proto-oncogenes (CDK4, MDM2, EGFR) activation [28, 29]. We found a poor association between primary brain tumor incidence and mEPHX exon 4 polymorphism (*χ* ^2^ = 4.3, *P* = 0.03, OR = 1.8%, 95 CI = 1.0−3.4) (Table 4). Thus, exon 4 polymorphism is probably protective against the risk of primary brain tumor in terms of exposure to PAH. There have been no reports about the relationship between brain tumors and mEPHX exon 4 polymorphism in the literature. The results of other studies, though, suggest a relationship between mEPHX exon 4 polymorphism and related cancer risks [30]. Wu et al. reported an increased risk of lung cancer among individuals with exon 4 (rapid allele), while there is a decreased risk of lung cancer among individuals with exon 3 (slow allele) [31]. No association between adenoma and mEH genotypes (exon 3 and 4) or mEH activity was observed by Cortessis et al. [32].

In this study, there was no statistically significant difference between cases and controls in smoking status (*χ* ^2^ = 3.5, *P* = 0.1) (Table 2). Our results suggest a minor role of tobacco smoking with brain tumor risk; nevertheless, an increased risk of adult glioma with smoking unfiltered cigarettes was observed by Lee et al. and Silvera et al. [33, 34]. Some carcinogenic compounds found in tobacco smoke cannot cross the blood-brain barrier, but some carcinogenic chemicals such as N-nitroso can cross it and may cause brain tumors [35, 36]. Several reactive metabolites in tobacco smoke may form adducts with DNA and cause carcinogenesis. The benzo (a) pyrene-7,8-dihydrodiol (BaP) is oxidized to benzo (a) pyrene-7,8-dihydrodiol 9,10-epoxide (BPDE), which binds to the exocyclic amino group of guanine DNA [37, 38].

We found a statistically significant association between brain tumor incidence and family history of cancer (*χ* ^2^ = 7.0, *P* = 0.01) (Table 3). This result is consistent with some other studies, suggesting that individuals with a family history of cancer may have an increased brain tumor risk [39, 40]. In one study, there were similar mechanisms for brain carcinogenesis, such as that of other organs [41].

In our case-control study, an increase in risk was not observed for any histological type of brain tumor associated with mEPHX variant genotypes. This finding is inconsistent with other studies and may be due to the small number of brain tumor cases in our study.

These results add to the literature about the contribution of variation in metabolic genes to the incidence of adult brain tumors. Overall, these data do not provide strong evidence for the importance of genes involved in biotransformation of PAHs and their metabolites for brain carcinogenesis; however, specific genes may play a role in the context of both PAHs and other exposures. We believe that the association between mEPHX enzyme polymorphisms and brain tumor incidence must be investigated by studies with a larger number of cases and controls.

In conclusion, our results somewhat support the hypothesis that genetic susceptibility to brain tumors may be associated with mEPHX gene polymorphisms. Additional research is still necessary to determine an individual's susceptibility to brain tumor development.

## Figures and Tables

**Table 1:** Characteristics of brain tumor cases and controls.

Characteristics	Controls	Cancer patients
Sample size	150	105
Gender		
Males	75 (50%)	55 (52%)
Females	75 (50%)	50 (48%)
Age (yr)		
Range	14–80	11–82
Males	50.0 ± 7.2	49.6 ± 16.3
Females	45.4 ± 15.8	47.5 ± 17.6
Smoking history (%)		
Smoker	38 (25%)	38 (36%)
Males	35 (92%)	30 (79%)
Females	3 (8%)	8 (21%)
Family history of cancer (%)	9 (6%)	17 (16%)

**Table 2:** Relationship between brain tumor patients and smoking status.

Smoking habit	Smokers	Nonsmokers
Cancer patients	38 (36%)	67 (64%)
Control	38 (25%)	112 (75%)
*χ* ^2^	3.5	
*P* value	0.1	

**Table 3:** Relationship between brain tumor patients and family history of cancer.

Family history of cancer	Yes	No
Cancer patients	17 (16%)	88 (84%)
Control	9 (6%)	141 (94%)
*χ* ^2^	7.0	
*P* value	0.01	

**Table 4:** Association between exon 3, exon 4 polymorphisms, and brain tumor.

Genotype	Cancer patients	Control	Crude OR (95% CI)
Exon 3			
Tyr/Tyr	58 (57%)	83 (55%)	1.0 (ref.)
Tyr/His, His/His	44 (43%)	67 (45%)	1.0 (0.6–1.7)
*χ* ^2^	0.05	
*P* value	0.8	

Exon 4			
His/His	85 (81%)	104 (69%)	1.0 (ref.)
His/Arg, Arg/Arg	20 (19%)	46 (31%)	1.8 (1.0–3.4)
*χ* ^2^	4.3	
*P* value	0.03	

**Table 5:** HWE for polymorphisms in mEH exon 3 and 4.

	Control		Patients	
Genotypes	Observed	Expected	Observed	Expected
Exon 3				
Homozygote reference	83	81.4	58	57.4
Heterozygotes	55	58.2	37	38.3
Homozygotes variant	12	10.4	7	6.4
Variance allele frequency	0.26		0.25	
*χ* ^2^ test *P* value (if < 0.05 not consistent with HWE)	0.79		0.94	

Exon 4				
Homozygote reference	104	106.7	85	85.1
Heterozygotes	45	39.6	19	18.9
Homozygotes variant	1	3.7	1	1.1
Variance allele frequency	0.16		0.10	
*χ* ^2^ test *P* value (if < 0.05 not consistent with HWE)	0.25		0.99	

**Table 6:** Association between mEPHX gene variants and histologic type of brain tumor.

Histologic type	Meningioma	Astrocytoma	H. adenoma	Other
Exon 3	30 (29%)	39 (38%)	9 (9%)	24 (24%)
Tyr/Tyr	19 (63%)	23 (59%)	5 (55%)	11 (46%)
Tyr/His, His/His	11 (37%)	16 (41%)	4 (45%)	13 (54%)
*χ* ^2^	0.4	0.05	0.01	0.9
*P* value	0.5	0.8	1.0*	0.3
OR (95% CI)	0.7 (0.3–1.9)	0.9 (0.4–2.0)	1.0 (0.2–4.8)	1.5 (0.5–4.1)

Exon 4	32 (30%)	39 (37%)	9 (9%)	25 (24%)
His/His	27 (84%)	33 (85%)	9 (100%)	16 (64%)
His/Arg, Arg/Arg	5 (16%)	6 (15%)	—	9 (36%)
*χ* ^2^	0.2	0.3	2.1	3.3
*P* value	0.7	0.6	0.3*	0.1
OR (95% CI)	0.7 (0.2–2.5)	0.7 (0.2–2.2)	—	2.4 (0.8–6.8)

*Fisher exact.

## Abbreviations

mEH: Microsomal epoxide hydrolase
PCR: Polymerase chain reaction
RFLP: Restriction fragment length polymorphism
OR: Odds ratio
CI: Confidence interval
PAHs: Polycyclic aromatic hydrocarbons
HWE: Hardy-Weinberg equilibrium.
